# Treatment preferences of patients with relapsed or refractory multiple myeloma in the United States, United Kingdom, Italy, Germany, France, and Spain: results from a discrete choice experiment

**DOI:** 10.3389/fmed.2023.1271657

**Published:** 2023-11-23

**Authors:** Caitlin Thomas, Sikander Ailawadhi, Rakesh Popat, David Kleinman, Melissa M. Ross, Boris Gorsh, Sarah Mulnick, Alicia O’Neill, Prani Paka, Maya Hanna, Nicolas Krucien, Alexa Molinari, Heather L. Gelhorn, Sue Perera

**Affiliations:** ^1^Patient-Centered Research, Evidera, London, United Kingdom; ^2^Divisions of Hematology-Oncology and Cancer Biology, Mayo Clinic, Jacksonville, FL, United States; ^3^University College London Hospitals NHS Foundation Trust, London, United Kingdom; ^4^Department of Ophthalmology, Flaum Eye Institute, University of Rochester Medical Center, Rochester, NY, United States; ^5^Patient-Centered Research, Evidera, Bethesda, MD, United States; ^6^GSK, Upper Providence, PA, United States; ^7^GSK, Philadelphia, PA, United States; ^8^Rutgers Center for Health Outcomes, Policy, and Economics, Rutgers, The State University of New Jersey, Piscataway, NJ, United States; ^9^GSK, London, United Kingdom

**Keywords:** benefit–risk, discrete choice experiment, multiple myeloma, patient preferences, trade-offs, treatment attributes

## Abstract

**Introduction:**

Newer treatment options for relapsed/refractory multiple myeloma (RRMM) with efficacy and safety profiles that differ from traditional therapies have facilitated personalized management strategies to optimize patient outcomes. In the context of such personalized management, understanding how treatment characteristics influence patients’ preferences is essential. This study assessed patients’ preferences for RRMM treatment attributes and determined trade-offs between potential benefits, administration procedures, and adverse effects.

**Methods:**

Patients’ preferences were evaluated using a discrete choice experiment (DCE). Patients with RRMM who reported failing two lines of anti-myeloma treatment (immunomodulatory agent and a proteasome inhibitor [PI]) or ≥ 3 lines (including ≥1 PI, immunomodulatory agent, or anti-CD38 monoclonal antibody), were recruited across the US, UK, Italy, Germany, France, and Spain. DCE attributes and levels were identified using a targeted literature review, a review of clinical data for relevant RRMM treatments, qualitative patient interviews, and input from clinical and myeloma patient experts. The DCE was administered within an online survey from February–June 2022. Preference data were analyzed using an error-component logit model and willingness to make trade-offs for potential benefits, and relative attribute importance scores were calculated.

**Results:**

Overall, 296 patients from the US (*n* = 100), UK (*n* = 49), Italy (*n* = 45), Germany (*n* = 43), France (*n* = 39), and Spain (*n* = 20) participated in the DCE. Mean (standard deviation) age was 63.8 (8.0) years, 84% had a caregiver, and patients had a median of 3 (range: 2–8) prior lines of therapy. Efficacy attributes most influenced patients’ preferences, with increasing overall response rate (25–85%) and overall survival (6 months to 2 years) contributing to ~50% of treatment decision-making. Administration procedures were also considered important to patients. Avoiding individual side effects was considered relatively less important, with patients willing to tolerate increases in side effects for gains in efficacy. Patient characteristics such as rate of disease progression, sociodemographics, or clinical characteristics also influenced treatment preferences.

**Conclusion:**

Patients with RRMM were willing to tolerate increased risk of side effects for higher efficacy. Preferences and risk tolerance varied between patients, with preference patterns differing by certain patient characteristics. This highlights the importance of shared decision-making for optimal treatment selection and patient outcomes.

## 1 Introduction

Multiple myeloma (MM) is an incurable hematological malignancy characterized by hypercalcemia, anemia, bone disease, and immunodeficiency (1). The global incidence of MM increased from 65,940 to 155,688 cases between 1990 and 2019 and is projected to increase by a further 51.3% by 2040 due in part to aging populations and higher diagnosis rates (2, 3). Among all cancers, patients with MM undergoing treatment are reported to have the poorest quality of life (4); despite improvements in therapeutic options, patients experience treatment-related adverse events and carry a heavy symptomatic burden. An improved understanding of MM pathobiology has driven advances in the management of MM through the development of novel treatments (5, 6). This progress has led to prolonged disease control and an improved overall 5-year relative survival rate (7, 8). Despite these advances, nearly all patients eventually relapse and become refractory to existing treatments, necessitating subsequent and numerous lines of treatment throughout their disease course (9, 10).

The availability of newer treatment options has facilitated personalized management strategies to optimize outcomes but also has led to an increasingly complex treatment pathway (11, 12). As a result, physicians need to balance efficacy and safety with assessment of patients’ needs and related considerations, such as those associated with treatment administration procedures, when considering therapeutic options (13). Shared decision-making is complex but essential, as it can inspire confidence in clinicians, improve patient adherence to treatments, and build the patient’s trust in the healthcare system (14, 15). How patients value different treatment characteristics is therefore an important consideration to aid shared decision-making, especially as additional treatment options become available and treatment decisions become more complex.

Previous studies have reported that survival or increased life expectancy, improved emotional quality of life, prolonged remission/response, reduced fatigue, and reduced worry are important benefits of treatment to patients with MM, whereas peripheral neuropathy, diarrhea/constipation, and cognitive impairment were noted as important side effects to avoid (13, 16–18). A few studies have examined preferences of patients with relapsed/refractory MM (RRMM); however, they were restricted regionally and did not assess preferences related to treatments that may have ocular side effects (13, 19).

The primary objective of the current study was to quantify patients’ preferences for RRMM treatment attributes and to calculate willingness to make trade-offs for potential benefits using a discrete choice experiment (DCE). The secondary objective was to assess heterogeneity of patients’ preferences based on clinical and sociodemographic characteristics with the aim of providing healthcare practitioners (HCPs) with information that may facilitate and inform shared decision-making when discussing treatment options with patients.

## 2 Materials and methods

### 2.1 Study population

Eligible patients were at least 18 years of age and had a self-reported diagnosis of RRMM with a self-reported treatment history of either (1) failing at least two lines of anti-myeloma treatments including an immunomodulatory agent and a proteasome inhibitor (PI), or (2) at least three lines of anti-myeloma treatments including at least one of a PI, an immunomodulatory agent, or an anti-CD38 monoclonal antibody. Additionally, patients were required to be a resident of the US, United Kingdom (UK), Italy, Germany, France, or Spain, and able to understand, read, and speak either English, Italian, German, French, or Spanish. Patients were invited using Institutional Review Board (IRB)-approved invitations and recruited by Global Perspectives, a specialized recruitment vendor. Patients were recruited by way of referrals from HCPs, patient associations, and social media. Informed consent was obtained from all patients online using a form approved by the IRB.

### 2.2 Study overview

**Figure 1:**
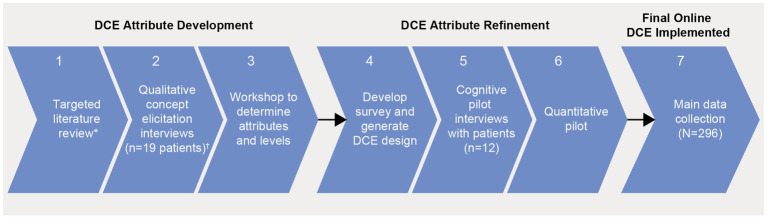
Study flow chart.*Published evidence on approved or developing treatments for RRMM, qualitative and quantitative preference studies; ^†^US, UK, Germany each *n* = 5 patients and France *n* = 4 patients. Clinical expert input was received for steps 1, 3, and 4; patient advisors from MM PEC input for steps 3, 4, and 5; advocacy groups input was received for steps 3 and 4. Input from clinical experts, patient advisors, and advocacy groups was received for analysis of the main data collection. DCE, discrete choice experiment; MM PEC, multiple myeloma patient expert council; RRMM, relapsed/refractory multiple myeloma.

An online survey containing a DCE was conducted in the United States (US) and Europe between February and June 2022. A DCE aims to simulate a scenario in which a patient may be informed about different treatment options by their physician. DCEs involve a series of questions that present patients with a choice between two or more hypothetical treatment options where they are forced to trade-off between different treatment attributes, such as administration, efficacy, and risk of adverse events. The attributes and levels included in the DCE (Table 1) were informed by an extensive process including a targeted literature review (TLR), expert clinical recommendations, and qualitative concept elicitation interviews (Figure 1). Attributes were also reviewed and refined by myeloma patient experts in the US and by Myeloma Patients Europe (MPE). The survey introduced the participants to each of the DCE attributes and gathered information related to patients’ preferences, as well as information related to sociodemographic, health-related quality of life (HRQoL), and clinical aspects of treatment.

**Table 1:** Final DCE attributes and levels.

**Attribute**	**Levels**
**Likelihood of responding** **to treatment**	**25%/40%/55%/70%/85%**
**Length of time in response (if you respond)**	**3 months/6 months/9 months/1 year/1.25 years**
**Lifespan**	**6 months/1 year/1.5 years/2 years**
**Tingling or pain in hands and/or feet** **(peripheral neuropathy)**	**0%/25%/50%**
**Temporary vision change**	**0%/** **20%** (20% mild, 0% moderate)/ **40%** (20% mild, 20% moderate)/ **60%** (40% mild, 20% moderate)
**Inflammatory response** **(CRS)**	**High risk** (15% do not experience, 80% have non-severe side effects, 5% have severe side effects)/ **No risk**
**Severe diarrhea**	**0%/10%/20%**
**Administration**	**IV or SC outpatient twice per week** until progression/ **IV or SC outpatient every 3 weeks** until progression/ **IV or SC outpatient every week + oral pills** until progression/ **IV or SC outpatient every month + oral pills** until progression **CAR-T therapy** Takes 1–2 months—one-time treatment until progression Inpatient in hospital for 7 days after treatment for monitoring Must stay near hospital for 4 weeks for monitoring after treatment Caregiver support required

CAR-T, chimeric antigen receptor T-cell; CRS, cytokine release syndrome; DCE, discrete choice experiment; IV, intravenous; SC, subcutaneous.

The study was conducted in accordance with European Medicines Agency guidelines on good pharmacovigilance practices (15), preference-based methods guidance from International Society for Pharmacoeconomics and Outcomes Research Good Practices for Outcomes Research, and applicable regulatory and country-specific requirements (20).

### 2.3 Attributes and level development

The TLR identified 17 publications (quantitative papers [*n* = 7]; qualitative papers [*n* = 10]). Topics related to patients’ preferences for MM treatment that emerged within the TLR fell into five categories, including efficacy, side effects, symptoms, treatment convenience, and QoL impact (Supplementary Table S1). Clinical efficacy and safety data of relevant RRMM treatments were extracted to determine whether there were any key treatment differences in efficacy and tolerability in the third line or later (3 L+) setting and to provide data for subsequent level development. The treatment combinations considered in the performance review included belantamab mafodotin (21, 22); idecabtagene vicleucel (23); melflufen and dexamethasone (24); selinexor and dexamethasone (25); pomalidomide and dexamethasone (26); carfilzomib and dexamethasone (27); isatuximab, pomalidomide and dexamethasone (28); elotuzumab, pomalidomide and dexamethasone (29); carfilzomib, pomalidomide with dexamethasone (30); panobinostat, bortezomib and dexamethasone (31); daratumumab, bortezomib and dexamethasone (32) where data were available. Administration procedures were also extracted for each treatment (Supplementary Table S2).

Qualitative concept elicitation interviews with 19 patients (US, UK, Germany, each *n* = 5; France, *n* = 4) were then conducted to confirm or refute the relevance and comprehensiveness of the treatment attributes identified in the TLR from the patient’s perspective, as well as identify any other potentially relevant treatment attributes not identified in the TLR (33). The results from the TLR, treatment performance extraction, and qualitative interviews were discussed with clinical experts during an attribute-selection workshop to ensure the selected attributes were clinically relevant to the decision context. Patient-centric focus was provided by MPE advocacy group and GSK’s internal standing patient advisory board (Multiple Myeloma Patient Expert Council [MM PEC]). The MPE and MM PEC were involved in determining attribute inclusion in the DCE, attribute discussions and provided feedback on patient-facing study materials to ensure all attribute definitions, as well as the visual presentation of attributes and levels, were patient-friendly and patient-driven.

The final attributes selected included three benefit attributes (likelihood of responding to treatment [ORR], length of time in response [DOR], and lifespan [OS]), four risk attributes (tingling pain in hands and/or feet [peripheral neuropathy], temporary vision change, severe diarrhea, and inflammatory response [cytokine release syndrome]), and mode of administration (defined above). The definitions of each attribute presented to the patients are described in Supplementary Table S4. For each attribute the level range was selected to encompass all potential outcomes of relevant RRMM treatments, for instance, cover the minimum level of benefit or side effect to the maximum level of benefit or side effect expected. Administration levels were selected to reflect the modes of administration for the majority of the RRMM treatments of interest. However, due to the variability of administrations available for RRMM treatments (Supplementary Table S2) it was not possible to include all potential levels.

### 2.4 Pilot testing and survey refinement

In January 2020, 60-min cognitive pilot interviews were conducted with two patients from each of the six countries to evaluate the feasibility and robustness of the DCE (total *n* = 12). Cognitive pilot interviews were conducted via web conference with synchronized screen sharing of survey materials between patients and interviewers, and were audio recorded with the patients’ permission. Interviews documented patient feedback in a spreadsheet and any aspect of the survey that was deemed difficult for patients to understand was revised. Key changes to the survey after interviews are provided in the Supplementary Table S3. These changes were made to ensure that attributes and levels were clear, comprehensive, and understandable to patients. Next, a soft-launch of the study was conducted for analyzing data after the first 77 patients were enrolled to assess whether the DCE was working as expected. No changes to the experimental design or survey were required based on the soft-launch findings.

### 2.5 DCE design

**Figure 2:**
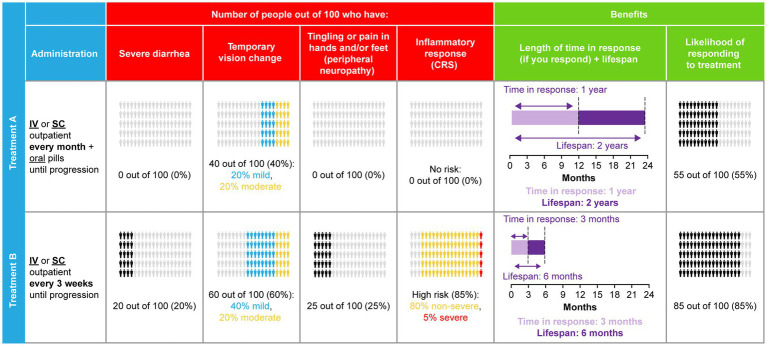
DCE sample choice task.CRS, cytokine release syndrome; DCE, discrete choice experiment; IV, intravenous; SC, subcutaneous.

The combinations of attribute levels shown for each hypothetical treatment option, within each choice task in a DCE, were generated with a D-efficient experimental design to ensure the choice tasks collected the maximum amount of information about tradeoffs between the attributes (34–36). The experimental design was generated using Ngene software (version 1.1.2, ChoiceMetrics, Sydney, Australia). The DCE design consisted of 36 choice tasks grouped into three blocks of 12 tasks. Patients were randomized to one of the three blocks to limit the cognitive burden of the DCE survey. Across the choice tasks, patients were repeatedly asked to choose between two mutually exclusive hypothetical treatment alternatives (Treatment A or Treatment B) with different levels of benefits/risks and modes of administration (Figure 2). Attributes were randomized within attribute groups (for example, benefit-, risk-, and administration-related) and attribute groups were randomized to mitigate the influence of ordering effects on preferences (37). After selecting their preferred option in the forced choice question, patients were given the possibility to opt-out, by indicating that they would not have taken either treatment if offered it by their doctor.

In addition to these 12 experimental choice tasks, patients also completed two internal validity choice tasks: stability and dominance tests. The stability test repeated experimental choice task 3, as seen by the patient, to assess whether patients were consistent in their choices, for instance, whether patients chose the same option as they had selected previously. The dominance test assessed patients’ engagement in the survey by assessing whether patients chose the superior (dominant due to higher efficacy and lower risks) option as preferred treatment.

### 2.6 Data analyses

Sociodemographics, clinical characteristics, and validity measures were reported descriptively. Patients’ treatment preferences were analyzed using an error-component logit (ECL) model within the random utility maximization framework. This model estimates the patients’ sensitivities to marginal changes in the treatment attributes, also referred to as marginal utilities, relative to a reference level. These effects were estimated using maximum likelihood-based estimation procedures (higher maximum likelihood estimates indicate a greater impact of that attribute level on preferences and indicate a more desirable change from the reference level). The estimated marginal utilities were then used to compute scores of relative attribute importance (RAI). RAI scores are conditional on the range of attribute levels, sum to 100%, and show the contribution of each attribute to treatment preferences. Further details of the DCE data analysis are provided in the Supplementary Methods.

Heterogeneity in patients’ preferences was then investigated by modeling the effects of differences in personal characteristics on patients’ sensitivities to changes in the attributes. This was achieved by adding interaction terms between the attributes’ levels and personal characteristics in the ECL model.

## 3 Results

### 3.1 Patient characteristics

In total, 296 patients completed the DCE (US, *n* = 100; UK, *n* = 49; Italy, *n* = 45; Germany, *n* = 43; France, *n* = 39; Spain, *n* = 20) (Table 2). Of the final DCE sample, median age was 65 (range: 38–85) years and 52% were male. Among patients in the US, 27% were White and 41% were Black/African American; 96.0% of UK patients were White, 2% were Asian, and 2% other. A significant portion of patients (16%) preferred not to state their race. In line with local research guidelines, race data were not collected in Italy, Germany, France, and Spain. At the time of survey, most patients (82%) lived with others, 84% had a caregiver (most commonly a family member, spouse, or partner [78%], friend or others [4%], or professional [1%]), and 56% were retired. Forty percent of the patients had received a maximum of primary or high school education, 20% attained partial college/university education, and 40% accomplished a college/university or postgraduate degree. All patients had some type of health coverage; the European countries had national healthcare or employer-provided/private health insurance (*n* = 296; 100%). While in the US, approximately half had Medicare (*n* = 49; 49%), the federal health insurance provided to seniors (≥65 years), and more than one third had private insurance (*n* = 37; 37%) (Table 2).

Patients were initially diagnosed with MM at a median of 5 (range: 1–28) years before the time of survey administration and had received a median of 3 (range: 2–8) prior anti-myeloma treatment (Table 2). Most patients reported having achieved a partial (46%) or complete (31%) anti-tumor response to treatment at the time of completing the survey. When asked to reflect on their HRQoL over the previous week, 22% of patients reported severe to very severe cancer-related symptoms, 28% of respondents reported severe to very severe pain, and 44% of patients reported severe to very severe fatigue (Table 3). Questions about steroid use were added after recruitment began; 47% of patients who answered (n = 120/258) had used steroids as part of their RRMM treatment history.

**Table 2:** Demographic and clinical characteristics by country.

		**Individual countries (% of overall population)**					
	**Overall** **(*N* = 296)**	**US** ***n* = 100 (34%)**	**UK** ***n* = 49** **(17%)**	**Italy** ***n* = 45** **(15%)**	**Germany** ***n* = 43** **(15%)**	**France** ***n* = 39** **(13%)**	**Spain** ***n* = 20** **(7%)**
**Age**, mean (SD), years	63.8 (8.0)	63.1 (4.9)	60.4 (8.9)	67.4 (5.9)	64.7 (9.7)	65.3 (10.4)	63.2 (10.2)
**Male**, *n* (%)	154 (52)	56 (56)	23 (47)	25 (56)	23 (53)	20 (51)	7 (35)
**Race**, *n* (%)*							
White^†^	74 (50)	27 (27)	47 (96)	–	–	–	–
Black^†^	41 (28)	41 (41)	0	–	–	–	–
Other^†^	10 (7)	8 (8)	2 (4)	–	–	–	–
Prefer not to say^†^	24 (16)	24 (24)	0	–	–	–	–
Not applicable*	147 (50)	0	0	45 (100)	43 (100)	39 (100)	20 (100)
**Ethnic background**, *n* (%)							
Hispanic or Latino^‡^	17 (17)	17 (17)	–	–	–	–	–
Not Hispanic or Latino^‡^	62 (62)	62 (62)	–	–	–	–	–
Prefer not to say^‡^	21 (21)	21 (21)	–	–	–	–	–
Missing	196 (66)	0	49 (100)	45 (100)	43 (100)	39 (100)	20 (100)
**Insurance status**, *n* (%)^§^							
Private insurance	92 (31)	37 (37)	6 (12)	3 (7)	3 (7)	37 (95)	6 (30)
Medicare	49 (17)	49 (49)	0	0	0	0	0
Medicaid	9 (3)	9 (9)	0	0	0	0	0
Veteran’s affairs	5 (2)	5 (5)	0	0	0	0	0
No private insurance	141 (48)	0 (0)	43 (88)	42 (93)	40 (93)	2 (5)	14 (70)
**Time since initial MM diagnosis**, median (range), years	5 (1–28)	5.4 (1.1–11.5)	6.5 (1.1–28)	2.5 (1–7.3)	4.3 (1.1–12.9)	7.3 (2.2–18.8)	5.3 (1.3–14.9)
**Time since first treatment for MM**, median (range), years	4.7 (0.3–28)	5.1 (1.1–11.7)	6.4 (1.1–28)	2.4 (1–7.4)	3.8 (1.1–12.9)	6.8 (0.3–18.9)	4.7 (1.3–15)
**Number of prior anti-myeloma treatments**, median (range)	3 (2–8)	3 (2–7)	3 (2–8)	3 (2–6)	4 (3–6)	4 (3–6)	4 (2–7)
**Response status**, *n* (%)							
Partial response	135 (46)	7 (7)	32 (65)	42 (93)	20 (47)	26 (67)	8 (40)
Complete response	92 (31)	36 (36)	13 (27)	3 (7)	23 (53)	8 (21)	9 (45)
Not in response	69 (23)	57 (57)	4 (8)	0	0	5 (13)	3 (15)
**Steroid experience**, *n* (%)							
Yes	120 (47)	24 (28)	32 (94)	5 (12)	30 (71)	10 (29)	19 (100)
No	88 (34)	56 (64)	2 (6)	8 (19)	7 (17)	15 (44)	0
Not sure	50 (19)	7 (8)	0	29 (69)	5 (12)	9 (26)	0
Not applicable^¶^	38 (13)	13 (13)	15 (31)	3 (7)	1 (2)	5 (13)	1 (5)
Importance of a steroid-free treatment option**							
Mean (SD)^††^	6.91 (2.46)	7.54 (2.54)	7.62 (2.34)	6.60 (1.67)	5.53 (1.80)	6.00 (3.20)	7.67 (2.43)
Median (range)	7 (1–10)	8 (1–10)	8 (2–10)	7 (4–8)	5 (3–10)	7 (1–10)	8 (2–10)

*Collection of race data was not permitted in Italy, Germany, France, and Spain. ^†^Data refer only to the US and UK population (*n* = 149). ^‡^Data refer to only the US population. ^§^Private insurance could be employer provided or self-provided for US patients; most European patients had national healthcare. ^¶^Steroid-related questions were introduced in a survey amendment and were not applied to 38 patients. ^**^Questions related to steroid experience were added after fielding had begun; therefore only 258/296 patients were shown the questions. ^††^1 = not at all important and 10 = extremely important. MM, multiple myeloma; SD, standard deviation.

**Table 3:** Current symptoms and health-related quality of life by country.

		**Individual countries (% of overall population)**					
	**Overall** **(*N* = 296)**	**US** ***n* = 100 (34%)**	**UK** ***n* = 49** **(17%)**	**Italy** ***n* = 45** **(15%)**	**Germany** ***n* = 43** **(15%)**	**France** ***n* = 39** **(13%)**	**Spain** ***n* = 20** **(7%)**
**Overall severity of cancer symptoms, *n* (%)**							
No symptoms	41 (14)	21 (21)	11 (22)	0	2 (5)	6 (15)	1 (5)
Mild–moderate	189 (64)	60 (60)	33 (67)	15 (33)	38 (88)	28 (72)	15 (75)
Severe–very severe	66 (22)	19 (19)	5 (10)	30 (67)	3 (7)	5 (13)	4 (20)
**Frequency of severe diarrhea in last 7 days, *n* (%)**							
Never	95 (32)	54 (54)	23 (47)	0	7 (16)	8 (21)	3 (15)
Rarely–occasionally	147 (50)	37 (37)	19 (39)	17 (38)	32 (74)	29 (74)	13 (65)
Frequently–almost constantly	54 (18)	9 (9)	7 (14)	28 (62)	4 (9)	2 (5)	4 (20)
**Severity of numbness/tingling in last 7 days, *n* (%)**							
None	87 (29)	53 (53)	13 (27)	0	11 (26)	5 (13)	5 (25)
Mild–moderate	158 (53)	41 (41)	30 (61)	22 (49)	27 (63)	27 (69)	11 (55)
Severe–very severe	51 (17)	6 (6)	6 (12)	23 (51)	5 (12)	7 (18)	4 (20)
**Severity of blurred vision in last 7 days, *n* (%)**							
None	114 (39)	51 (51)	22 (45)	0	20 (47)	11 (28)	10 (50)
Mild–moderate	150 (51)	46 (46)	27 (55)	25 (56)	18 (42)	26 (67)	8 (40)
Severe–very severe	32 (11)	3 (3)	0	20 (44)	5 (12)	2 (5)	2 (10)
**Severity of pain in last 7 days, *n* (%)**							
None	54 (18)	24 (24)	17 (35)	0	2 (5)	6 (15)	5 (25)
Mild–moderate	160 (54)	43 (43)	26 (53)	19 (42)	32 (74)	29 (74)	11 (55)
Severe–very severe	82 (28)	33 (33)	6 (12)	26 (58)	9 (21)	4 (10)	4 (20)
Severity of fatigue, tiredness, or lack of energy in last 7 days, *n* (%)							
None	19 (6)	8 (8)	7 (14)	0	0	3 (8)	1 (5)
Mild–moderate	146 (49)	48 (48)	28 (57)	4 (9)	27 (63)	30 (77)	9 (45)
Severe–very severe	131 (44)	44 (44)	14 (29)	41 (91)	16 (37)	6 (15)	10 (50)

### 3.2 Patient preferences for RRMM treatments attributes

**Figure 3:**
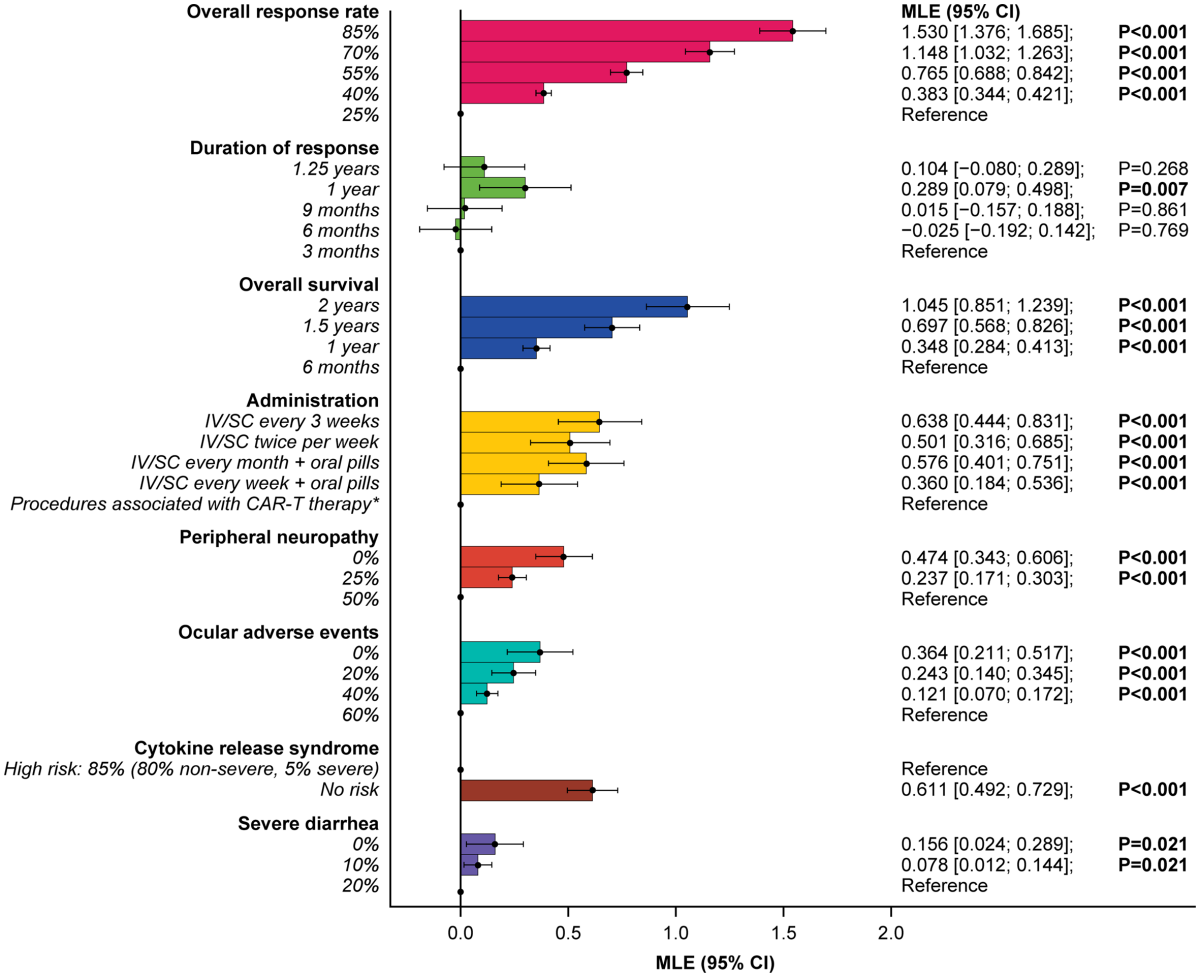
Patient preferences for treatment attribute levels.Reference indicates the level to which each utility is compared. *Administration procedures associated with CAR-T therapy were described to participants as follows: Takes 1–2 months—one-time treatment until progression; inpatient in hospital for 7 days after treatment for monitoring; must stay near hospital for 4 weeks for monitoring after treatment; caregiver support required. CAR-T, chimeric antigen receptor T-cell; CI, confidence interval; IV, intravenous; MLE, maximum likelihood estimate; SC, subcutaneous.

**Figure 4:**
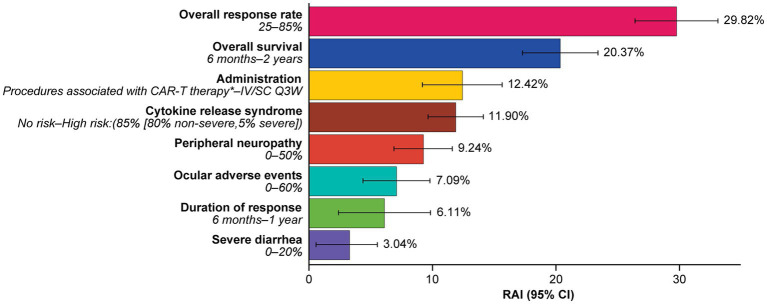
Relative attribute importance scores for treatment attributes.*Administration procedures associated with CAR-T therapy were described to participants as follows: Takes 1–2 months—one-time treatment until progression; inpatient in hospital for 7 days after treatment for monitoring; must stay near hospital for 4 weeks for monitoring after treatment; caregiver support required. RAI scores capture the maximum contribution of each attribute to a treatment preference in the DCE. RAI scores are conditional on the range of attribute levels and sum to 100%. Information in parenthesis refers to the range of levels analyzed. CAR-T, chimeric antigen receptor T-cell; CI, confidence interval; DCE, discrete choice experiment; IV, intravenous; Q3W, every 3 weeks; RAI, relative attribute importance; SC, subcutaneous.

At least one level for each attribute was significant, and estimated preferences were in the expected direction for most ordered attributes (i.e., preference for higher efficacy and lower levels of risks). Attributes related to efficacy were the most important consideration for patients, with increasing ORR from 25 to 85% (RAI: 29.8%) and OS from 6 months to 2 years (RAI: 20.4%) having the greatest influence on patients’ preferences for RRMM treatment (both *p* < 0.001) (Figure 3) and accounting for half (50.2%) of the total RAI (Figure 4).

Administration procedures (RAI: 12.4%) were ranked third in terms of RAI, although relative importance did not differ notably from some other lower-ranked attributes, with considerable overlap of 95% confidence intervals (CI) observed. On average, IV or subcutaneous (SC) treatment every 3 weeks without pills, and IV or SC twice a week without pills, were preferred over a treatment with one-time administration associated with CAR-T therapy (described as a process that takes 1 month [one-time treatment until progression] and requires staying as an inpatient in a hospital for 7 days after treatment for monitoring; patients would be required to stay near the hospital for 4 weeks for monitoring after treatment and caregiver support would be required) (Figure 3).

Avoiding side effects in general was one of the least important considerations to patients. In RAI rank order, patients preferred to avoid CRS (RAI: 11.9%; 95% CI [9.7, 14.1]), peripheral neuropathy (RAI: 9.2%; 95% CI [6.9, 11.6]), ocular adverse effects (RAI: 7.1%; 95% CI [4.4, 9.8]), and severe diarrhea (RAI: 3.0%; 95% CI [0.5, 5.5]) (Figure 4).

### 3.3 Minimal acceptable benefit

**Figure 5:**
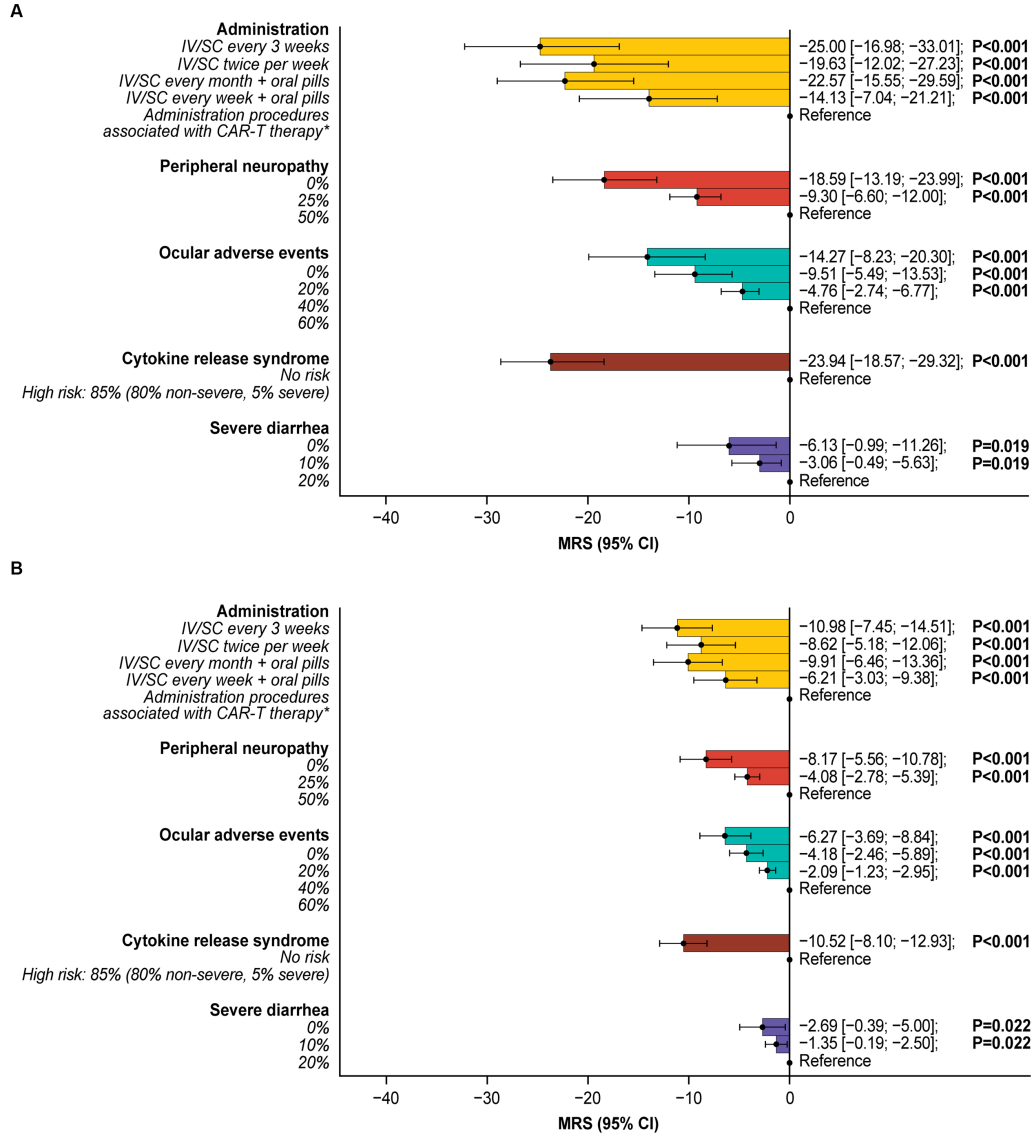
Incremental marginal rates of substitution for **(A)** overall response rate and **(B)** overall survival.*Administration procedures associated with CAR-T therapy were described to participants as follows: Takes 1–2 months—one-time treatment until progression; inpatient in hospital for 7 days after treatment for monitoring; must stay near hospital for 4 weeks for monitoring after treatment; caregiver support required. All MRS calculations are relative to the reference level. For example, patients were willing to tolerate a 60% risk of ocular adverse events (over 0% risk) for a 14.27% higher ORR or an additional 6.27 months’ OS. CAR-T, chimeric antigen receptor T-cell; CI, confidence interval; IV, intravenous; MRS, marginal rate of substitution; ORR, overall response rate; OS, overall survival; SC, subcutaneous.

The minimal acceptable benefit (MAB) estimates indicated that patients were willing to make trade-offs between side effects and efficacy, tolerating increased side-effect risks in exchange for higher efficacy, in terms of higher ORR and longer OS. To tolerate an 85% risk of CRS (80% non-severe, 5% severe; Supplementary Table S4), a 60% risk of ocular adverse events, a 50% risk of peripheral neuropathy, and a 20% risk of severe diarrhea, patients would require a 23.9%, 14.3%, 18.6%, and 6.1% increase in ORR, respectively. Similarly, for OS, patients would require an additional 10.5, 6.3, 8.2, and 2.7 months of OS for the same risks, respectively (Figure 5). To be willing to undergo a treatment with administration procedures associated with CAR-T therapy rather than IV/SC administration every 3 weeks, patients would require a 25% increase in ORR or an additional 11.0 months of OS.

### 3.4 Impact of personal characteristics on treatment preferences

Preferences for treatment attributes varied by patients’ sociodemographic and clinical characteristics including but not limited to region, age, caregiver status, MM response status, number of prior lines of therapy, ocular side-effect experience, and fatigue severity (Supplementary Table S5). In the US, almost two-thirds of the population were not in response (*n* = 57; 57%), whereas around one-third were in complete response (*n* = 36; 36%) while few were in partial response (*n* = 7; 7%) (Table 2). US patients (*n* = 100; 34%) placed a greater relative importance on increasing ORR and DOR and preferred less-frequent IV or SC administration (with or without oral pills) to more-frequent administration or administration procedures associated with CAR-T therapy. However, in Europe (*n* = 196; 66%) a greater relative importance was placed on increasing OS and avoiding ocular adverse events and CRS (Supplementary Figure S1). Older patients (≥60 years [*n* = 220; 74%]) preferred IV/SC administration (with or without pills) over administration procedures associated with CAR-T therapies and placed a greater relative importance on avoiding peripheral neuropathy (Supplementary Figure S2). While patients with a caregiver (*n* = 248; 84%) placed greater relative importance on increasing ORR and administration aspects, with a preference for less-frequent IV/SC administration (with or without oral pills), patients without a caregiver (*n* = 48; 16%) placed a greater relative importance on increasing OS and avoiding ocular adverse events and peripheral neuropathy (Supplementary Figure S3). Patients not in response (*n* = 69; 23%) placed greater importance on increasing ORR and avoiding administration procedures associated with CAR-T therapy than patients in complete (*n* = 92; 31%) or partial response (*n* = 135; 46%) (Supplementary Figure S4). Patients earlier in their treatment pathway (2–3 prior lines of therapy [*n* = 176; 59%]) placed greater relative importance on increasing ORR compared with those on later lines of therapy (4 or more lines of treatment [*n* = 120; 40.5%]). Patients who received 5 or more lines of treatment (*n* = 43; 15%) preferred administration procedures associated with CAR-T therapy, whereas patients who received fewer lines of therapy (*n* = 253; 85% [2–4 lines of therapy]) preferred treatments that did not involve administration procedures associated with CAR-T therapy (Supplementary Figure S5). Patients who had never experienced blurry vision (*n* = 114; 39%) placed greater relative importance on increasing ORR (Supplementary Figure S6). Patients not experiencing fatigue or those with moderate fatigue (*n* = 165; 56%) placed greater importance on increasing OS and avoiding CRS than patients with more severe fatigue (*n* = 131; 44%) (Supplementary Figure S7).

## 4 Discussion

This robust, quantitative patient preference study, exploring the opinions of patients with RRMM, found that treatment preferences were strongly driven by maximizing treatment efficacy (ORR and OS), compared with other treatment characteristics related to therapeutic administration methods and risk of specified side effects. Patients would be willing to tolerate increased risks of burdensome side effects and complex administration procedures (including CAR-T therapy and different combinations of oral pills, IV or SC injections) for a treatment associated with adequate improvements in efficacy. Although patients wanted to avoid major side effects associated with anti-myeloma treatment, including CRS, peripheral neuropathy, ocular adverse effects, and severe diarrhea, there was a willingness to tolerate increased risks of these side effects for improved anti-tumor response rates and OS. For instance, patients would tolerate a treatment with a 60% increase in the risk of ocular adverse events for an increase in ORR by 14.3% or an increase in OS of 6.3 months.

These findings on the importance of efficacy aligns with other patient preferences studies. One stated preference study of 560 patients with MM found that most respondents (58%) placed greater importance on increasing the probability of being progression-free for 1 year or longer than simultaneously decreasing the probability of severe or life-threatening toxicity and mild or moderate chronic toxicity (38). In another DCE study, among 94 patients with RRMM and 32 caregivers in the US, it was found that longer progression-free survival and avoidance of severe nerve damage were the most important to patients (13).

Aspects associated with administration procedures in general were also important to patients, with less-frequent IV/SC treatments preferred over the administration procedures associated with CAR-T therapy. Nonetheless, patients assessed in this study were willing to accept administration procedures associated with CAR-T therapy over IV or SC administration every 3 weeks for improved efficacy (25% increase in ORR or an additional 11.0 months of OS). Similarly, another DCE study of 84 patients that evaluated patients’ preferences for treatment in MM reported that mode of administration was the most important attribute among PI-based combination treatments (19).

Although a measure of efficacy, DOR was less important to patients than the other efficacy-related attributes. Patients had a clear preference to increase DOR from 3 months to 1 year; however, significant preferences for other levels were not detected. However, as the DCE presented the DOR attribute in a combined timeline with OS, part of the value of DOR is captured within OS. Therefore, an exploratory model specification with OS and DOR in an alternate format was generated and found that DOR was important to patients, with a preference for longer DOR. However, as part of DOR was captured within OS in the original analysis, the specific preference for DOR may not be obvious when presented as part of OS.

Different preferences for treatment attributes were associated with various sociodemographic and clinical characteristics including, among others, region, age, MM response status, and prior lines of therapy. Similarly, in another DCE study involving 475 patients with MM, patients recently diagnosed (in the last 5 years) placed greater importance on efficacy (survival) than those diagnosed more than 5 years ago. In accordance with this study, patients who underwent longer treatment placed greater importance on mode of administration than those treated for a shorter period (39). Preferences of patients with 5 or more prior lines of therapy preferring CAR-T therapy aligns with CAR-T being indicated for those patients and may signify a desire to try a new type of treatment after failing several prior lines.

As with all DCE studies, several factors and limitations should be considered when interpreting the results. Patients’ choices were restricted because they were required to make decisions based solely on the information provided. Furthermore, choices were made in isolation and considering all other things to be equal, which may not reflect clinical practice, whereby input is obtained from multiple individuals, including doctors, family members, and caregivers. To limit the cognitive burden of the DCE survey, only the most relevant and important treatment attributes were included; hence the scores of relative importance are conditional on the list of attributes included in the study and should not be extrapolated to other treatment aspects. Given the wide variety of administration procedures for RRMM treatments, along with the methodological and cognitive burden constraints that limit the number of levels that can be included in a DCE, an all-oral administration level was not included. While few RRMM treatments involve an all-oral regimen, patients may prefer this route, as was suggested in a recent study (19).

While this study focused on the preferences of patients from countries with established healthcare systems, it is difficult to generalize patients’ preferences beyond the countries in this study. Also, although sample sizes were too small to analyze preference heterogeneity between individual countries, the differences between the US and Europe were explicitly compared, however, these differences may also be linked to other correlated variables such as response status. An additional limitation could be the potential selection bias wherein patients’ preferences within this study may be systematically different from the general population with RRMM.

In the survey, patients were provided with attribute definitions and were asked to make hypothetical treatment decisions based on the information they had available without discussing options with their clinician. A disadvantage of using an online survey is that not all patients may have access to computers and thus the sample may not be representative of the overall RRMM population. However, due to increased familiarity with the internet even among older patients, this is becoming less of a concern. In clinical practice, physicians can monitor and manage risks such as CRS and ocular adverse events and might be able to alleviate patients’ concerns by discussing potential monitoring and management strategies as part of shared decision-making. Although this study included patients with self-reported diagnoses of MM, the inclusion of rigorous screening eligibility questions designed to assess RRMM lines of treatment within the survey mitigated against the inclusion of ineligible patients. Finally, as the DCE is based on hypothetical decisions, a disparity between stated choices and choices that patients would make in real-life situations may exist. However, the hypothetical choice tasks were designed to mimic these real-life situations as closely as possible.

## 5 Conclusion

With significant progress in treatment options for MM as well as novel therapies now in development for patients in recent years, the treatment decision-making process for patients can be complex. Patients with RRMM who had received at least two lines of prior therapy including both an immunomodulatory agent and a PI, or at least three lines of anti-myeloma treatments including at least one of a PI, an immunomodulatory agent, or an anti-CD38 monoclonal antibody were more focused on increasing efficacy, with less importance placed on reducing the risk of specific side effects. Increasing ORR and OS were considered to be the most important aspects of treatment, and patients were also willing to tolerate increased risks of side effects in exchange for efficacy gains in ORR and OS. Overall, patients were least concerned about avoiding severe diarrhea. Differences in preferences were identified, with varied treatment priorities, based on sociodemographic and clinical characteristics. The results of this study highlight the importance of a holistic needs assessment and shared decision-making, with clear communication about the potential risks and benefits of available treatments, to ensure an understanding of the needs and desires of the patients, based on their individual situation.

## Data availability statement

The datasets generated and/or analyzed during the study are not publicly available as consent was not sought from participants to allow sharing of data with third parties.

## Ethics statement

The study was approved by Salus IRB (study number: 20201) and all participants provided online consent before participating in the study. This study was conducted in accordance with the guidelines on good pharmacovigilance practices (European Medicines Agency, 2014), preference-based methods from International Society for Pharmacoeconomics and Outcomes Research (ISPOR), Good Practices for Outcomes Research (International Council for Harmonisation of Technical Requirements for Pharmaceuticals for Human Use, 1996), and applicable regulatory and country-specific requirements.

## Author contributions

CT: Conceptualization, Data curation, Formal analysis, Writing – original draft, Writing – review & editing, Methodology. SA: Formal analysis, Writing – original draft, Writing – review & editing, Methodology. RP: Formal analysis, Writing – original draft, Writing – review & editing, Methodology. DK: Formal analysis, Writing – original draft, Writing – review & editing, Methodology. MR: Conceptualization, Data curation, Formal analysis, Writing – original draft, Writing – review & editing, Methodology. BG: Formal analysis, Writing – original draft, Writing – review & editing, Methodology. SM: Conceptualization, Data curation, Formal analysis, Writing – original draft, Writing – review & editing, Methodology. AO’N: Formal analysis, Writing – original draft, Writing – review & editing, Methodology. PP: Formal analysis, Writing – original draft, Writing – review & editing, Methodology. MH: Conceptualization, Writing – original draft, Writing – review & editing, Methodology. NK: Formal analysis, Writing – original draft, Writing – review & editing. AM: Formal analysis, Writing – original draft, Writing – review & editing, Methodology. HG: Methodology, Formal analysis, Writing – original draft, Writing – review & editing, Methodology. SP: Formal analysis, Writing – original draft, Writing – review & editing, Methodology.
