# Supplementary material for: Treatment preferences of patients with relapsed or refractory multiple myeloma in the United States, United Kingdom, Italy, Germany, France, and Spain: results from a discrete choice experiment
Source: Front Med (Lausanne). 2023 Nov 23;10:1271657. doi: 10.3389/fmed.2023.1271657 (PMC10702501; doi:10.3389/fmed.2023.1271657)
Supplement: Supplementary file 1 [file Data_Sheet_1.docx]

# **SUPPLEMENTARY MATERIAL**

## Supplementary methods:

## Discrete choice analyses

The analysis of all discrete choice experiment (DCE) data followed random utility maximization theory, which assumes that DCE participants always chose the alternative that results in the highest utility (a measure of desirability), based on the included attributes and up to a random error.

### *Modeling preference heterogeneity*

An error component multinomial logit (ECL) model was estimated to test for panel effects (i.e., multiple choice observations coming from the same participant) in the data. The ECL model with interaction terms between the personal characteristics of the participant and the attribute levels was used to assess heterogeneity based on observable characteristics of the participant (e.g., age).

Several personal characteristics were included in the analysis of preference heterogeneity. To be included in the analysis, each characteristic must have represented at least 10% of the sample size. The overall effect of a personal characteristic on preferences was evaluated by allowing for all possible interaction effects between the personal characteristic and attributes’ levels. The overall effect of the personal characteristic on preference heterogeneity was measured with the change in statistical performance relative to the reference model. The superiority of the model with interaction terms was evaluated with a log-likelihood ratio test. A significant test indicates an effect of the personal characteristic on preferences.

## Behavioral outputs

### *Relative attribute importance*

Relative attribute importance (RAI) was calculated to measure the importance of each attribute relative to all other attributes conditional on the range of levels of that attribute. The difference in preference weights between the best or most preferred level of an attribute and the worst or least preferred level of the same attribute provided an estimate of the relative importance of that attribute over the range of levels included in the DCE.

### *Willingness to trade off*

Minimum acceptable benefit (MAB) was calculated using overall response rate (ORR) and overall survival (OS) to measure participants’ valuation of treatment attributes using a common unit (e.g., % for ORR and months for OS). MAB was calculated to understand the minimum increase in % ORR, or months of OS, that participants would need to be compensated with for increases in the risk of peripheral neuropathy, ocular side effects, cytokine release syndrome (CRS), and severe diarrhea as well as to accept an administration procedure associated with chimeric antigen receptor T-cell (CAR-T) therapy over intravenous (IV) or subcutaneous (SC) administration (with or without oral pills). MAB was obtained as the marginal rate of substitution between the marginal utility of each attribute and the linear coded parameter of ORR or OS.

## Supplementary Table 1. Attributes identified in the targeted literature review

|  | **Quantitative papers (n=7) + 1 quantitative abstract** | **Qualitative papers (n=10)** |
| --- | --- | --- |
| **Mode, frequency, and location of administration** | 6 | 5 |
| **Cost** | 2 | 5 |
| **Overall survival** | 5 | 6 |
| **Remission** | 6 | 2 |
| **Side effects** | 8 | 8 |
| **Tingling hands or feet (peripheral neuropathy)** | 1 | 4 |
| **Blood-associated side effects (e.g., anemia, clotting disorders, neutropenia, thrombocytopenia)** | 2 | 2 |
| **Physical QoL** | 2 | 2 |
| **Emotional QoL** | 2 | 3 |
| **Social QoL** | 2 | 1 |
| **General HRQoL** | 0 | 3 |
| **Therapy-free intervals** | 2 | 1 |
| **Bone pain** | 0 | 4 |
| **Independence** | 1 | 2 |

HRQoL, health-related quality of life; QoL, quality of life.

## Supplementary Table 2. Administration procedures for various treatment regimens

| **Treatment regimen** | **Mode of administration and schedule*** |
| --- | --- |
| Belantamab mafodotin (1, 2) | IV, every 3 weeks |
| Idecabtagene vicleucel (3) | one-time administration of CAR-T therapy |
| Melflufen; dexamethasone (4) | IV, once-monthly; oral, once-weekly |
| Selinexor and dexamethasone (5) | oral, twice weekly |
| Pomalidomide; dexamethasone (6) | oral, once daily; oral or IV, weekly |
| Carfilzomib and dexamethasone (7) | IV on days 1, 2, 8, 9, 15 and 16 per cycle |
| Isatuximab; pomalidomide; dexamethasone (8) | IV, 4x a month; oral, once daily until on days 1 to 21 in each cycle; oral or IV, 4x a month in each cycle |
| Elotuzumab; pomalidomide; dexamethasone (9) | IV, 4x during cycles 1 and 2; oral, once daily until day 21 of each cycle; oral, per week |
| Carfilzomib; pomalidomide; dexamethasone (10) | IV, 6x every 28 days for the first 6 cycles; oral, once-daily on days 1 to 21 every 28 days; oral or IV, 4x per 28-days |
| Panobinostat; bortezomib; dexamethasone (11) | oral, thrice per week on weeks 1 and 2; IV, twice per week on weeks 1 and 2; oral, 4x per week on weeks 1 and 2 |
| Daratumumab; bortezomib; dexamethasone (12) | IV, once per week during first 3 cycles; subcutaneously, 4x during cycles 1–8; oral or IV, 8x per cycle |

*Specific details pertaining to the treatment schedule can be found in the supporting references.

## Supplementary Table 3. Key changes made post cognitive pilot interviews

| **Topic** | **Changes made after cognitive pilots** |
| --- | --- |
| Screener | Updates to the screener to allow patients to build their line of therapy by treatment type and, if a treatment was not listed, to select ‘other’ and then build their combination therapy. Clarified that for a line of treatment to end, patients must have terminated all treatments within a combination. |
| Experimental design | Updated the constraints of duration of response and overall survival so that the two attributes were never shown as the same duration in the same choice task. |
| Introductory information | Created a visual diagram of the CAR-T administration process. |
| Survey length | Removed questions about choice certainty and difficulty after the DCE to reduce the length of the survey. |
| Follow-up opt-out question | Revised to ask patients to imagine that the two treatments were the only two treatments available, and then to answer whether they would have chosen to take the treatment that they selected or to take neither of the two treatments. |
| Response attribute wording | Changed ‘remission’ to ‘response’ to reflect clinical practice. Conversations with MMPEC advisors confirmed this was understood by patients across both the US and EU, including once translated. |

## Supplementary Table 4. Definition of attributes presented to patients

| **Attribute** | **Definition presented to participants** |
| --- | --- |
| **Likelihood of responding to treatment** | The percentage, or number of people out of 100, who respond to treatment.   - Responding to a treatment means that you have a reduction in the number of cancerous cells in your blood (M-proteins or paraproteins). - If you respond, the number of cancer cells is significantly reduced.   - You may have a partial response, where there are still some cancer cells left, or you may have a complete response, where the cancerous cells can no longer be detected. - The likelihood of responding (how many people out of 100 respond) is different between treatments. |
| **Length of time in response (if you respond)** | If you respond to treatment, the length of time you maintain this response varies between treatments. If you lose response or relapse, you may need to start on a new treatment. If you respond, you would have at least a partial response, where you have fewer cancer cells and experience fewer symptoms, or you could be in complete response, where your myeloma is no longer detectable.  If you lose response or relapse, you may need to start on a new treatment. |
| **Lifespan** | This is the length of time that you will be alive after starting a new treatment. |
| **Tingling or pain in hands and/or feet  (peripheral neuropathy)** | Some treatments can damage nerve cells (that carry messages between the brain and the body), which may lead to tingling in your hands and/or feet (peripheral neuropathy). You may experience sharp pain, a feeling of pins and needles, numbness, feeling pain or heat when touching something cold, have some pain when touching objects, and/or a lack of balance or coordination. The likelihood (risk) of experiencing damaged nerve cells and this feeling in your hands and/or feet varies between treatments.  If you experience peripheral neuropathy, you may have a temporary treatment break (or drug holiday), a dose reduction, or need to change treatment. |
| **Temporary vision change** | With some treatments you may experience a temporary change in your vision after about 2 months of treatment. If you experience a change in vision, on average, the expected time for your vision to recover is 4 to 5 weeks.  In very rare cases (fewer than 2 out of 100, or fewer than 2% of cases), you may experience a more severe impact on vision.   - If you experience a **mild temporary vision change**, you may notice a change in your vision, but it will not impact your daily activities. - If you experience a **moderate temporary vision change**, images will appear slightly blurry and you may experience dry eyes, which may be itchy. This may impact your ability to do some activities, such as driving or reading, but you will be able to continue with other daily activities.   If you experience temporary vision changes, you may have a temporary treatment break (or drug holiday), a dose reduction, or need to change treatment to allow your vision to recover and prevent further issues. |
| **Inflammatory response (CRS)** | Some treatments have a high risk of an inflammatory response (cytokine release syndrome [CRS]), whereas others do not. Inflammatory response (CRS) can be harmful and interfere with bodily functions, causing organ failure and, in rare instances, death (less than 1%).  In this study, you will be shown some treatments that have a high risk of an inflammatory response (CRS) and others that do not. Inflammatory response (CRS) usually lasts for around 5 days, but in rare cases it may last for up to 2 months.  If a treatment has a high risk of inflammatory response (CRS), of 100 patients on the treatment, the inflammatory response (CRS) experienced is summarized below:   - **15 out of 100 people (15%)** do not have inflammatory response (CRS) and/or side effects from inflammatory response (CRS). - **80 out of 100 people (80%)** have inflammatory response (CRS) that results in side effects that are not severe, such as fever, chills, fatigue, weakness, loss of appetite, nausea, vomiting, diarrhea, headache, joint or muscle aches, or skin rash. They may be given oxygen if they have difficulty breathing, or IV immunosuppressants to treat the inflammatory response (CRS). - **5 out of 100 people (5%)** have inflammatory response (CRS) that results in severe side effects, such as damage to the heart, brain, lungs, kidney, and/or liver. This is treated with IV steroids, as well as oxygen and immunosuppressants. In some cases, patients with severe side effects need to be put on a ventilator to support their breathing or given dialysis for damaged kidneys. **In rare cases (less than 1%) patients experience a very severe inflammatory response (CRS) that results in death.** |
| **Severe diarrhea** | Multiple myeloma treatments may cause severe diarrhea, but the chance of experiencing severe diarrhea depends on the treatment you are given. With severe diarrhea you will experience 7 or more watery stools per day. You are unlikely to be able to control your bowel movements and you may have trouble going about your daily activities. Severe diarrhea may not be controlled with over-the-counter medication and may require hospitalization with IV hydration. You may have to discontinue treatment that causes severe diarrhea.  If you experience severe diarrhea, you may have a temporary treatment break (or drug holiday), a dose reduction, or need to change treatment. |
| **Administration** | There are a variety of treatments available or being developed for MM. There are different ways of administering these treatments:   - Some treatments are **oral pills**, which can be taken at home. - Others require **IV** (into the vein) **infusions** or **SC** (injection under the skin and above the muscle) at the hospital as an outpatient. - Some require a **combination** of both **oral pills** at home and **IV infusions** or **SC injections** at the hospital, as an outpatient.   The **frequency** of the IV infusions or SC injections also **varies** from weekly to monthly. These treatments are used according to the dosing schedule until progression.  An alternative treatment is **CAR-T therapy.*** |

*CAR-T therapy was described as a one-time treatment until progression involving several steps over 1–2 months, followed by hospitalization for a week after treatment for monitoring, must stay near hospital for 4 weeks for monitoring post-treatment, and requiring continuous caregiver support for 2–8 weeks after discharge.

CAR-T, chimeric antigen receptor T-cell; CRS, cytokine release syndrome; IV, intravenous; MM, multiple myeloma; SC, subcutaneous.

## Supplementary Table 5. Summary of key differences in relative attribute importance due to observed characteristics

|  | | **ORR** | **DOR** | **OS** | **Peripheral neuropathy** | **Ocular adverse events** | **CRS** | **Severe diarrhea** | **Administration** |
| --- | --- | --- | --- | --- | --- | --- | --- | --- | --- |
| **Sociodemographic** | **Region** | US | US | Europe | - | Europe | Europe | - | US |
|  | **Age** | - | - | <60 years* | ≥60 years* | - | - | - | ≥60 years* |
|  | **Caregiver status** | Has caregiver* | - | Does not have a caregiver* | - | Does not have a caregiver* | - | - | Has a caregiver* |
|  | **Employment Status** | Not employed* | Employed^†^* | - | - | - | - | - | - |
|  | **Education** | - | - | University and higher* | - | - | - | - | Below university* |
|  | **Household income** | - | - | Higher income* | - | - | - | - | Lower income* |
|  | **Distance from treatment center** | <1 hour* | - | - | ≥1 hour away* | - | - | - | - |
| **Disease characteristics** | **Time since diagnosis** | - | - | ≤5 years ago* | - | - | - | - | - |
|  | **Number of prior lines of treatment** | 2–3 lines of treatment* | - | - | - | - | 4 lines of treatment* | - | - |
|  | **Response status** | Not in response* | - | Partial or complete response* | Partial response* | Partial response* | Partial response* | - | Not in response* |
| **Disease severity** | **Severity of cancer symptoms** | No symptoms* | - | Mild–moderate symptoms* | - | - | Mild–moderate symptoms* | - | - |
|  | **Impact on activity level** | - | - | Normal—normal activities*^†^ | - | Normal—normal activities*^†^ | Normal—normal activities*^†^ | In bed for less than half day*^‡^ | - |
|  | **Peripheral neuropathy experience** | None* | - | Mild–moderate* | Severe to very severe* | - | Mild–moderate * | - | - |
|  | **Prior experience of ocular side effects** | None* | - | - | - | - | - | - | - |
|  | **Pain severity** | - | - | - | - | - | Mild–moderate * | - | - |
|  | **Fatigue severity** | - | - | Mild–moderate * | - | - | - | - | - |

Europe includes United Kingdom, Italy, Germany, France, and Spain. Of 296 patients, 100 (34%) were from the US and 196 (66%) were from Europe (UK, n=49 [17%]; Italy, n=45 [15%]; Germany, n=43 [15%]; France, n=39 [13%]; Spain, n=20 [7%]).

*Indicates subgroups that had a significantly greater RAI for a given attribute. ^†^'Normal with no limitations' or 'do not feel like your normal self but are able to keep up with normal activities.'  ^‡^'Do not feel up to most things, in bed or chair for less than half a day.'

CRS, cytokine release syndrome; DOR, duration of response; ORR, overall response rate; OS, overall survival; RAI, relative attribute importance.

## Supplementary Figure 1. Impact of region on preferences (N=296)

**
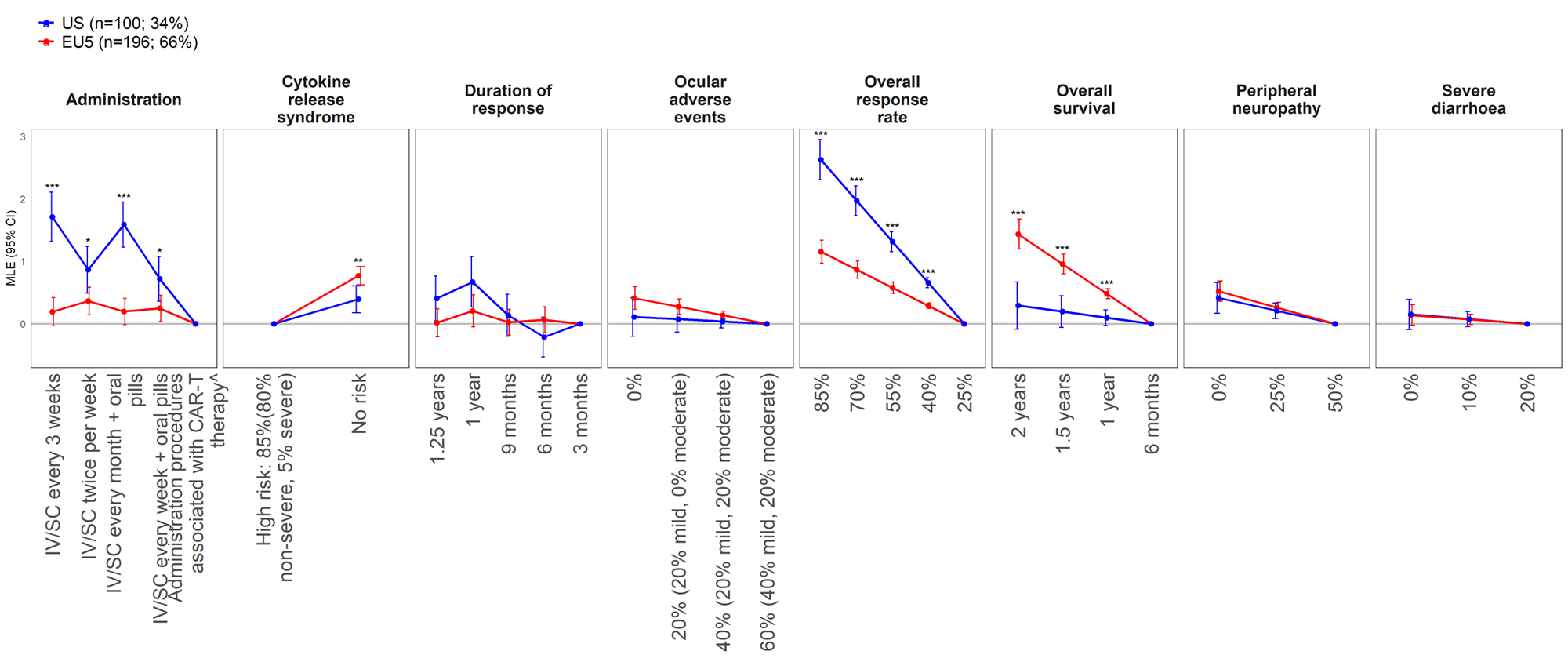
**

*p-value < 5%; **p-value < 1%; ***p-value < 0.1%; ^†^Administration procedures associated with CAR-T therapy were described to participants as follows: Takes 1–2 months – one-time treatment until progression; inpatient in hospital for 7 days after treatment for monitoring; must stay near hospital for 4 weeks for monitoring after treatment; caregiver support required.

CAR-T, chimeric antigen receptor T-cell; CI, confidence interval; IV, intravenous; MLE, maximum likelihood estimate; SC, subcutaneous; MLE, maximum likelihood estimate.

## Supplementary Figure 2. Impact of age on preferences (N=296)

**
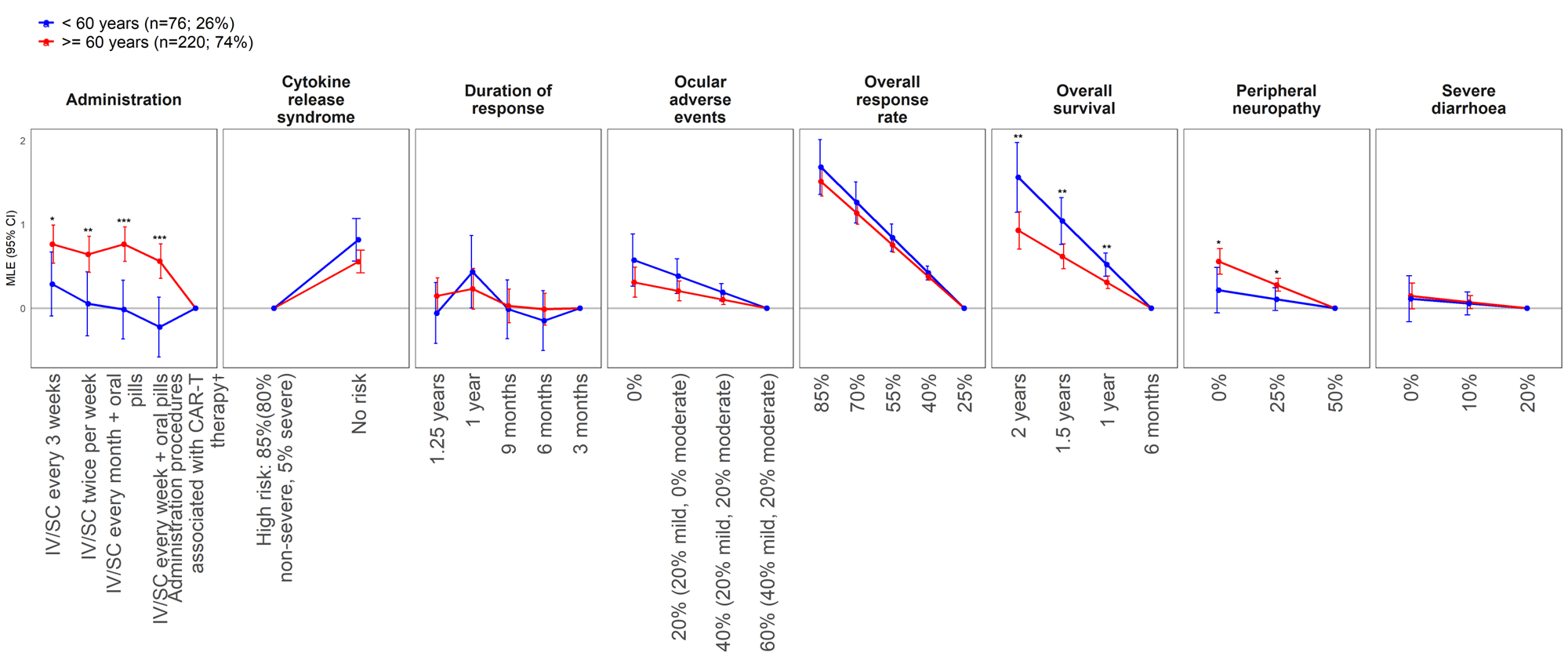
**

*p-value < 5%; **p-value < 1%; ***p-value < 0.1%; ^†^Administration procedures associated with CAR-T therapy were described to participants as follows: Takes 1–2 months – one-time treatment until progression; inpatient in hospital for 7 days after treatment for monitoring; must stay near hospital for 4 weeks for monitoring after treatment; caregiver support required.

CAR-T, chimeric antigen receptor T-cell; CI, confidence interval; IV, intravenous; MLE, maximum likelihood estimate; SC, subcutaneous; MLE, maximum likelihood estimate.

## Supplementary Figure 3. Impact of needing a caregiver on preferences (N=296)


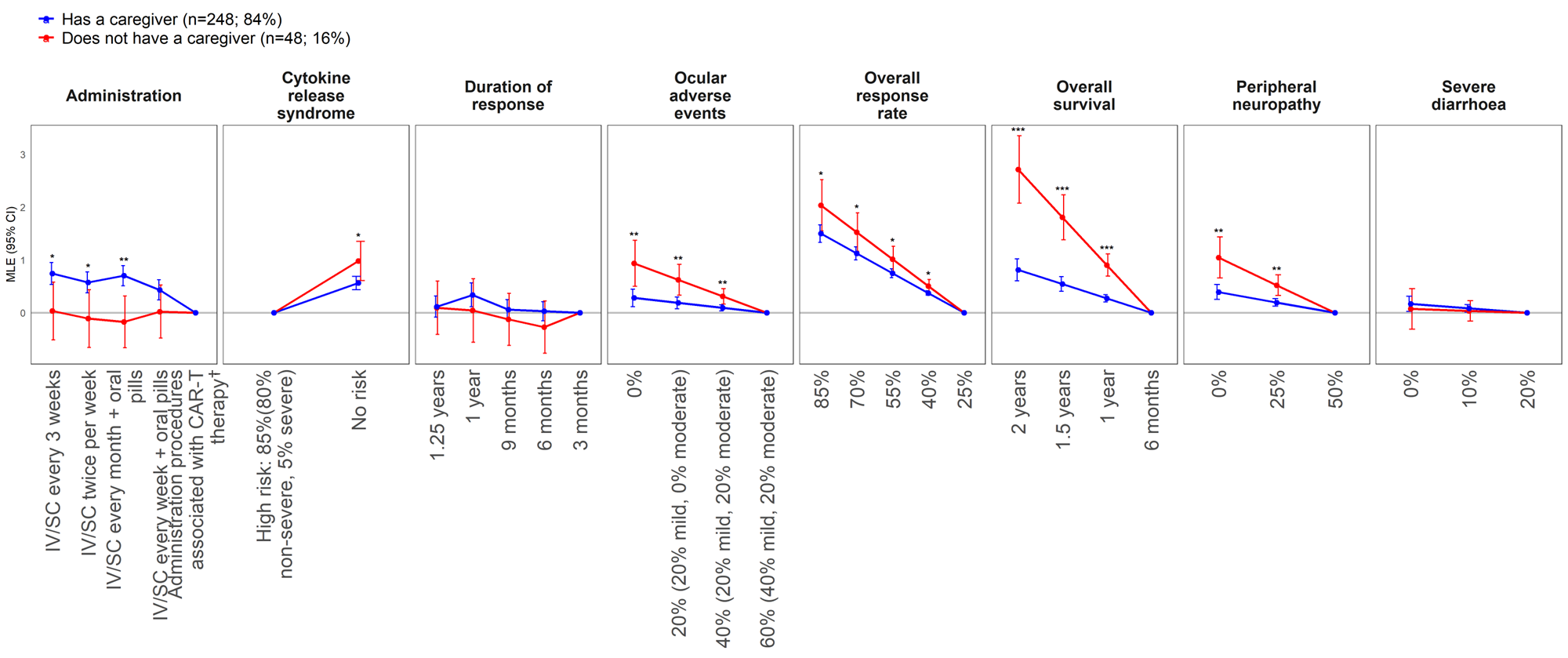


*p-value < 5%; **p-value < 1%; ***p-value < 0.1%; ^†^Administration procedures associated with CAR-T therapy were described to participants as follows: Takes 1–2 months – one-time treatment until progression; inpatient in hospital for 7 days after treatment for monitoring; must stay near hospital for 4 weeks for monitoring after treatment; caregiver support required.

CAR-T, chimeric antigen receptor T-cell; CI, confidence interval; IV, intravenous; MLE, maximum likelihood estimate; SC, subcutaneous; MLE, maximum likelihood estimate.

## Supplementary Figure 4. Impact of anti-tumor response on preferences (N=296)

**
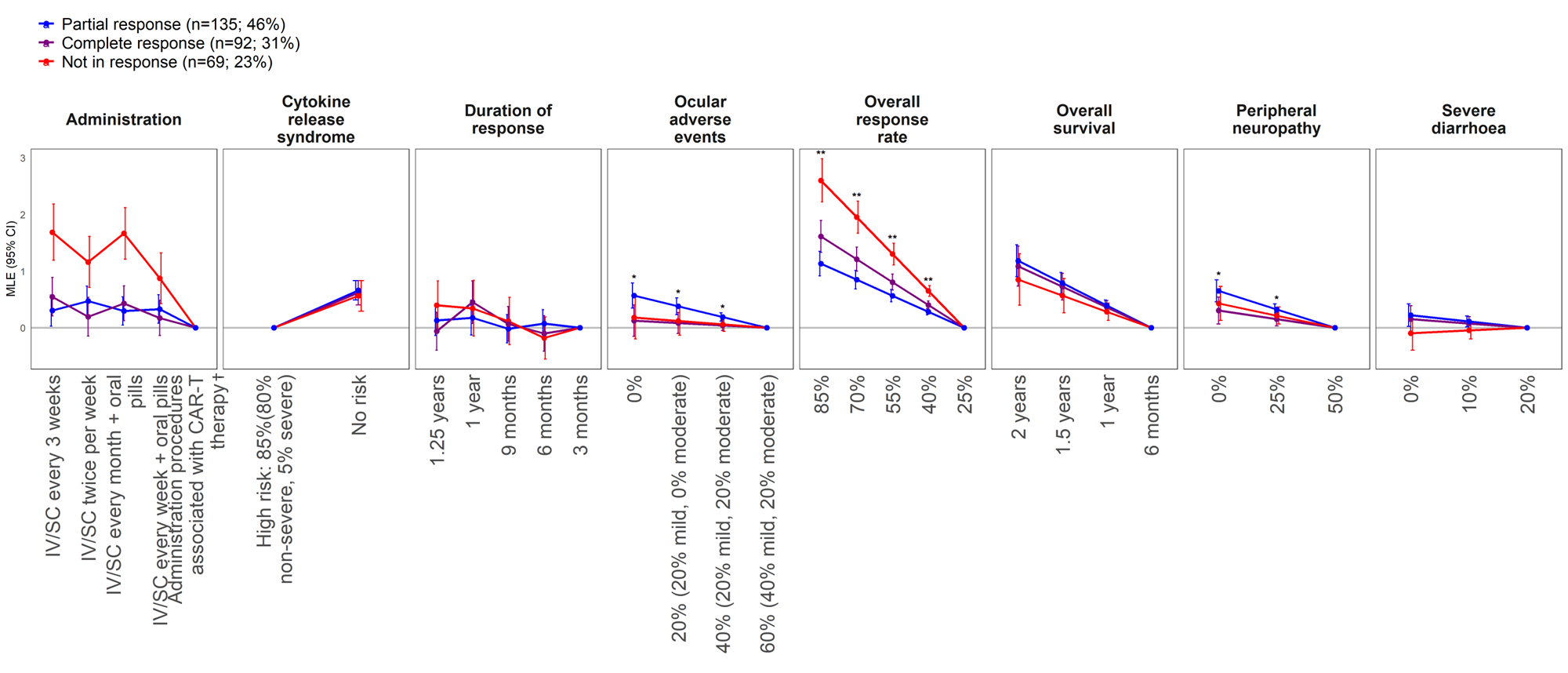
**

*p-value < 5%; **p-value < 1%; ***p-value < 0.1%; ^†^Administration procedures associated with CAR-T therapy were described to participants as follows: Takes 1–2 months – one-time treatment until progression; inpatient in hospital for 7 days after treatment for monitoring; must stay near hospital for 4 weeks for monitoring after treatment; caregiver support required.

CAR-T, chimeric antigen receptor T-cell; CI, confidence interval; IV, intravenous; MLE, maximum likelihood estimate; SC, subcutaneous; MLE, maximum likelihood estimate.

## Supplementary Figure 5. Impact of lines of prior therapy on preferences (N=296)

**
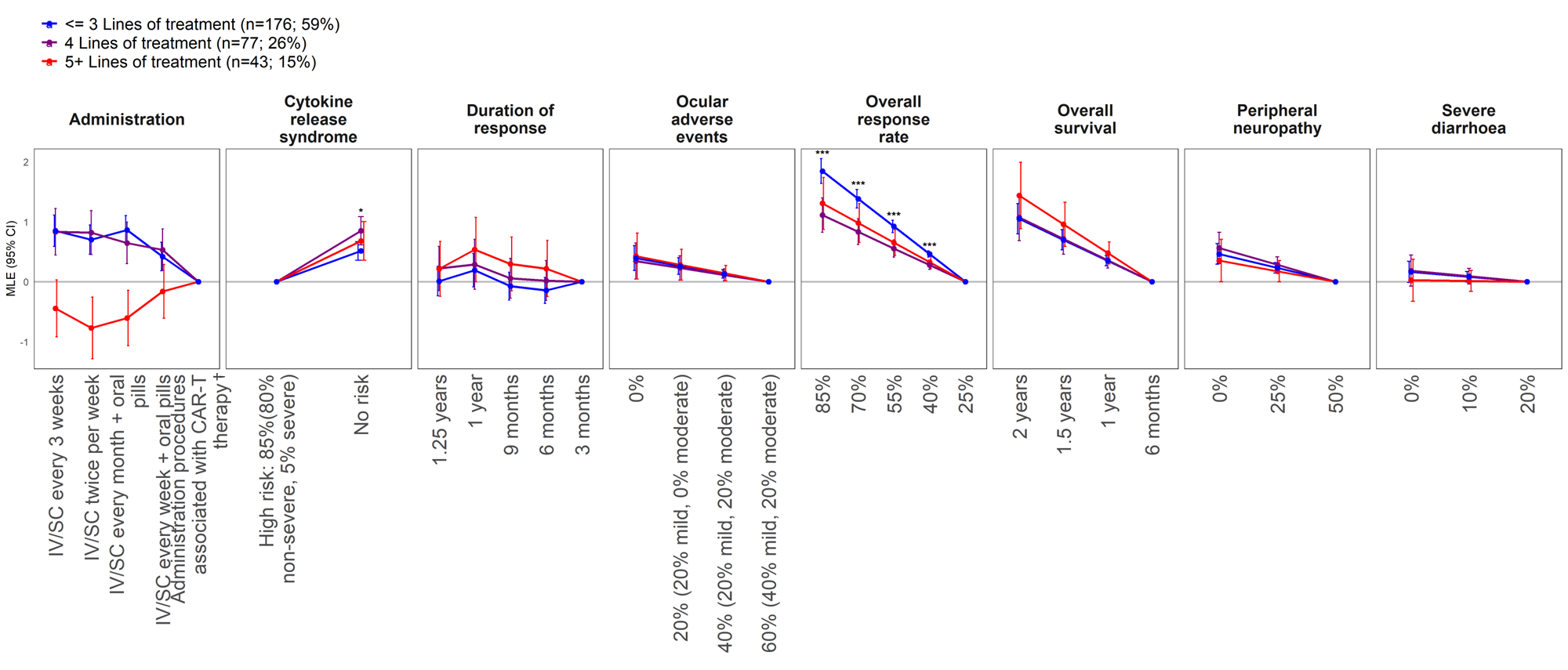
**

*p-value < 5%; **p-value < 1%; ***p-value < 0.1%; ^†^Administration procedures associated with CAR-T therapy were described to participants as follows: Takes 1–2 months – one-time treatment until progression; inpatient in hospital for 7 days after treatment for monitoring; must stay near hospital for 4 weeks for monitoring after treatment; caregiver support required.

CAR-T, chimeric antigen receptor T-cell; CI, confidence interval; IV, intravenous; MLE, maximum likelihood estimate; SC, subcutaneous; MLE, maximum likelihood estimate.

## Supplementary Figure 6. Impact of prior experience of blurry vision on preferences (N=296)

**
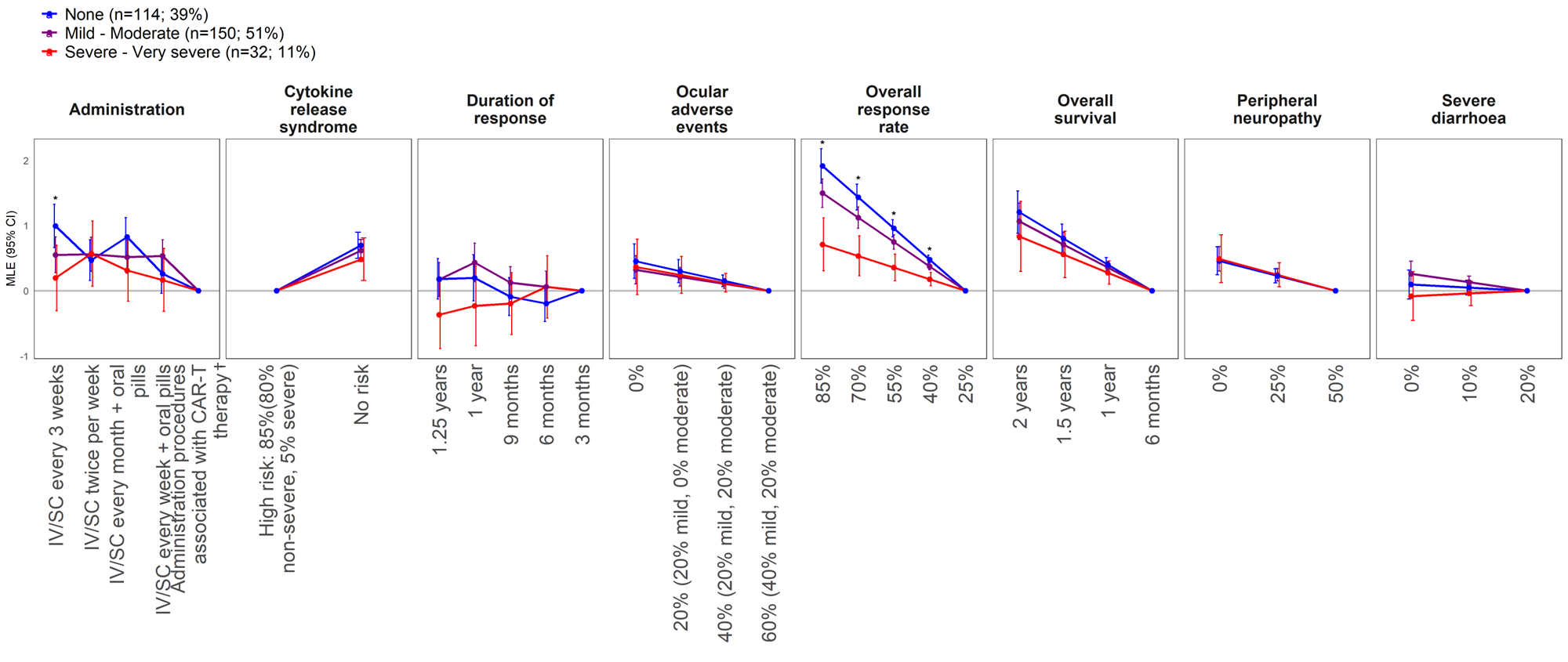
**

*p-value < 5%; **p-value < 1%; ***p-value < 0.1%; ^†^Administration procedures associated with CAR-T therapy were described to participants as follows: Takes 1–2 months – one-time treatment until progression; inpatient in hospital for 7 days after treatment for monitoring; must stay near hospital for 4 weeks for monitoring after treatment; caregiver support required.

CAR-T, chimeric antigen receptor T-cell; CI, confidence interval; IV, intravenous; MLE, maximum likelihood estimate; SC, subcutaneous; MLE, maximum likelihood estimate.

**Supplementary Figure 7. Impact of severity of prior fatigue experience on preferences (N=296)**


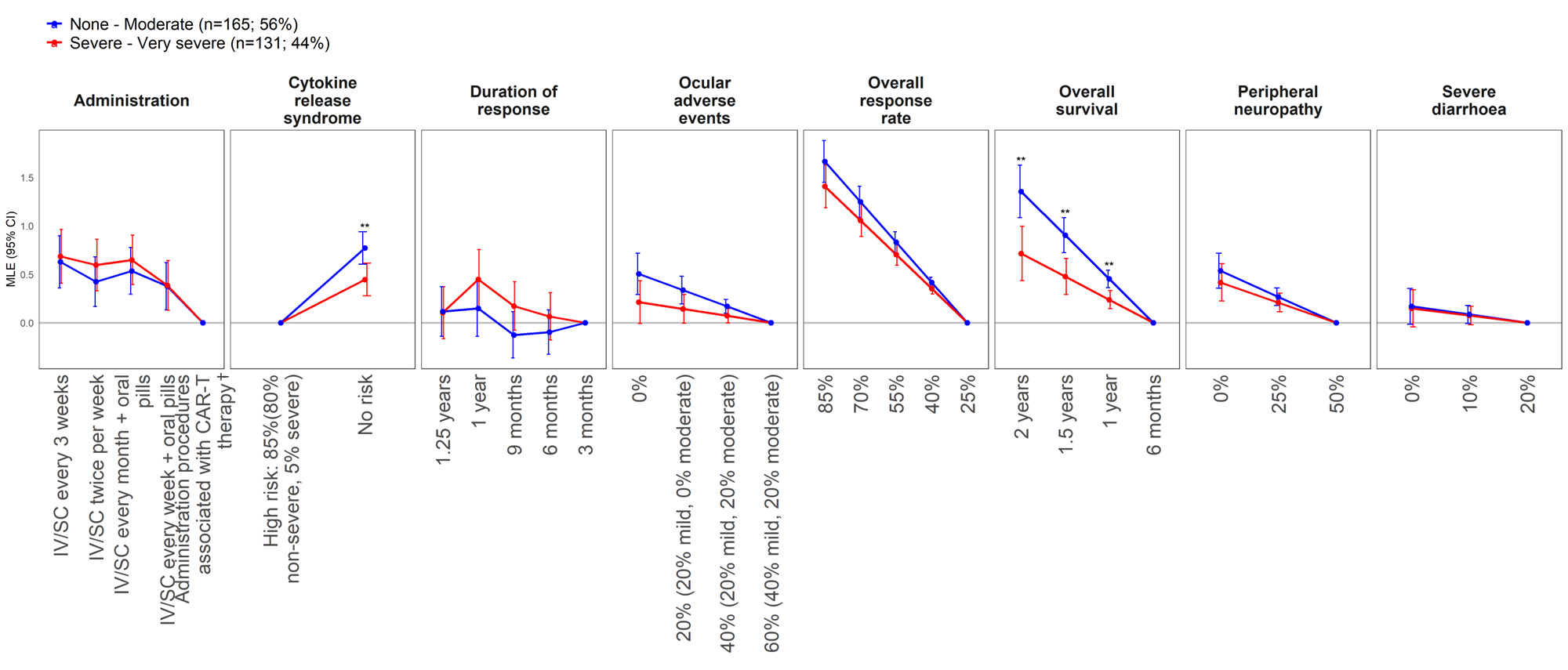


*p-value < 5%; **p-value < 1%; ***p-value < 0.1%; ^†^Administration procedures associated with CAR-T therapy were described to participants as follows: Takes 1–2 months – one-time treatment until progression; inpatient in hospital for 7 days after treatment for monitoring; must stay near hospital for 4 weeks for monitoring after treatment; caregiver support required.

CAR-T, chimeric antigen receptor T-cell; CI, confidence interval; IV, intravenous; MLE, maximum likelihood estimate; SC, subcutaneous; MLE, maximum likelihood estimate.

**References**

1. Lonial S, Lee HC, Badros A, Trudel S, Nooka AK, Chari A, et al. Belantamab Mafodotin for Relapsed or Refractory Multiple Myeloma (Dreamm-2): A Two-Arm, Randomised, Open-Label, Phase 2 Study. *The Lancet Oncology* (2020) 21(2):207-21. Epub 2019/12/21. doi: 10.1016/s1470-2045(19)30788-0.

2. Richardson PG, Lee HC, Abdallah AO, Cohen AD, Kapoor P, Voorhees PM, et al. Single-Agent Belantamab Mafodotin for Relapsed/Refractory Multiple Myeloma: Analysis of the Lyophilised Presentation Cohort from the Pivotal Dreamm-2 Study. *Blood cancer journal* (2020) 10(10):106. Epub 2020/10/25. doi: 10.1038/s41408-020-00369-0.

3. Munshi NC, Anderson LD, Jr., Shah N, Madduri D, Berdeja J, Lonial S, et al. Idecabtagene Vicleucel in Relapsed and Refractory Multiple Myeloma. *The New England journal of medicine* (2021) 384(8):705-16. Epub 2021/02/25. doi: 10.1056/NEJMoa2024850.

4. Richardson PG, Oriol A, Larocca A, Bladé J, Cavo M, Rodriguez-Otero P, et al. Melflufen and Dexamethasone in Heavily Pretreated Relapsed and Refractory Multiple Myeloma. *Journal of clinical oncology : official journal of the American Society of Clinical Oncology* (2021) 39(7):757-67. Epub 2020/12/10. doi: 10.1200/jco.20.02259.

5. Chari A, Vogl DT, Gavriatopoulou M, Nooka AK, Yee AJ, Huff CA, et al. Oral Selinexor-Dexamethasone for Triple-Class Refractory Multiple Myeloma. *The New England journal of medicine* (2019) 381(8):727-38. Epub 2019/08/23. doi: 10.1056/NEJMoa1903455.

6. Soekojo CY, Kim K, Huang SY, Chim CS, Takezako N, Asaoku H, et al. Pomalidomide and Dexamethasone Combination with Additional Cyclophosphamide in Relapsed/Refractory Multiple Myeloma (Amn001)-a Trial by the Asian Myeloma Network. *Blood cancer journal* (2019) 9(10):83. Epub 2019/10/09. doi: 10.1038/s41408-019-0245-1.

7. Ailawadhi S, Sexton R, Lentzsch S, Abidi MH, Voorhees PM, Cohen AD, et al. Low-Dose Versus High-Dose Carfilzomib with Dexamethasone (S1304) in Patients with Relapsed-Refractory Multiple Myeloma. *Clinical cancer research : an official journal of the American Association for Cancer Research* (2020) 26(15):3969-78. Epub 2020/04/18. doi: 10.1158/1078-0432.ccr-19-1997.

8. Attal M, Richardson PG, Rajkumar SV, San-Miguel J, Beksac M, Spicka I, et al. Isatuximab Plus Pomalidomide and Low-Dose Dexamethasone Versus Pomalidomide and Low-Dose Dexamethasone in Patients with Relapsed and Refractory Multiple Myeloma (Icaria-Mm): A Randomised, Multicentre, Open-Label, Phase 3 Study. *Lancet (London, England)* (2019) 394(10214):2096-107. Epub 2019/11/19. doi: 10.1016/s0140-6736(19)32556-5.

9. Dimopoulos MA, Dytfeld D, Grosicki S, Moreau P, Takezako N, Hori M, et al. Elotuzumab Plus Pomalidomide and Dexamethasone for Multiple Myeloma. *The New England journal of medicine* (2018) 379(19):1811-22. Epub 2018/11/08. doi: 10.1056/NEJMoa1805762.

10. Shah JJ, Stadtmauer EA, Abonour R, Cohen AD, Bensinger WI, Gasparetto C, et al. Carfilzomib, Pomalidomide, and Dexamethasone for Relapsed or Refractory Myeloma. *Blood* (2015) 126(20):2284-90. Epub 2015/09/19. doi: 10.1182/blood-2015-05-643320.

11. Richardson PG, Schlossman RL, Alsina M, Weber DM, Coutre SE, Gasparetto C, et al. Panorama 2: Panobinostat in Combination with Bortezomib and Dexamethasone in Patients with Relapsed and Bortezomib-Refractory Myeloma. *Blood* (2013) 122(14):2331-7. Epub 2013/08/21. doi: 10.1182/blood-2013-01-481325.

12. Palumbo A, Chanan-Khan A, Weisel K, Nooka AK, Masszi T, Beksac M, et al. Daratumumab, Bortezomib, and Dexamethasone for Multiple Myeloma. *The New England journal of medicine* (2016) 375(8):754-66. Epub 2016/08/25. doi: 10.1056/NEJMoa1606038.
